# Resistance Training Prevents Muscle Loss Induced by Caloric Restriction in Obese Elderly Individuals: A Systematic Review and Meta-Analysis

**DOI:** 10.3390/nu10040423

**Published:** 2018-03-29

**Authors:** Amanda V. Sardeli, Tiemy R. Komatsu, Marcelo A. Mori, Arthur F. Gáspari, Mara Patrícia T. Chacon-Mikahil

**Affiliations:** 1 Laboratory of Exercise Physiology—FISEX, Faculty of Physical Education, University of Campinas (UNICAMP), Campinas, Sao Paulo 13083-851, Brazil; arthur.fg@hotmail.com (A.F.G.); marapatricia@fef.unicamp.br (M.P.T.C.-M.); 2 Gerontology Program—Faculty of Medical Sciences, UNICAMP, Campinas, Sao Paulo 13083-887, Brazil; tiemy.komatsu@gmail.com; 3 Laboratory of Aging Biology (LaBE), Department of Biochemistry and Tissue Biology, Institute of Biology, University of Campinas (UNICAMP), Campinas, Sao Paulo 13083-862, Brazil; morima@unicamp.br; 4 Graduate Program in Genetics and Molecular Biology, Institute of Biology University of Campinas (UNICAMP), Campinas, Sao Paulo 13083-862, Brazil

**Keywords:** exercise, training, aging, sarcopenia, muscle mass, strength training, caloric restriction, diet

## Abstract

It remains unclear as to what extent resistance training (RT) can attenuate muscle loss during caloric restriction (CR) interventions in humans. The objective here is to address if RT could attenuate muscle loss induced by CR in obese elderly individuals, through summarized effects of previous studies. Databases MEDLINE, Embase and Web of Science were used to perform a systematic search between July and August 2017. Were included in the review randomized clinical trials (RCT) comparing the effects of CR with (CRRT) or without RT on lean body mass (LBM), fat body mass (FBM), and total body mass (BM), measured by dual-energy X-ray absorptiometry, on obese elderly individuals. The six RCTs included in the review applied RT three times per week, for 12 to 24 weeks, and most CR interventions followed diets of 55% carbohydrate, 15% protein, and 30% fat. RT reduced 93.5% of CR-induced LBM loss (0.819 kg [0.364 to 1.273]), with similar reduction in FBM and BM, compared with CR. Furthermore, to address muscle quality, the change in strength/LBM ratio tended to be different (*p* = 0.07) following CRRT (20.9 ± 23.1%) and CR interventions (−7.5 ± 9.9%). Our conclusion is that CRRT is able to prevent almost 100% of CR-induced muscle loss, while resulting in FBM and BM reductions that do not significantly differ from CR.

## 1. Introduction

Caloric restriction (CR) has been shown to increase lifespans and attenuate the harmful effects of aging across the evolutionary spectrum [1,2]. Retrospective studies also demonstrate an association between CR and health span in humans [3]. The exact mechanism underlying the benefits of CR remains unknown, but it involves changes in nutrient-sensing pathways, metabolic homeostasis, and body composition [1,4,5,6]. Weight loss is a normal feature of CR, and some groups claim that it is necessary for beneficial effects, including a reduction of chronic inflammation, which is an important trigger of non-communicable diseases [7,8,9]. However, weight loss via CR is accompanied by a significant decrease in lean body mass (LBM) [10], which may be deleterious to elderly individuals suffering from sarcopenia. Sarcopenia is often associated with frailty and increased mortality at advanced ages, and is a challenge for successful aging [11].

Resistance training (RT), associated or not with a high protein intake, has been shown to increase LBM, promote strength, and attenuate sarcopenia in elderly individuals [12,13]. However, it is unclear whether RT represents a good strategy to prevent muscle loss during CR (which includes dietary protein restriction). Some studies have reported no change or even reduced LBM following CR when RT is included in the intervention program [7,14,15,16,17,18]. On the other hand, randomized clinical trials (RCT) comparing CR with and without RT have shown the preservation of LBM with RT [19,20]. This inconsistency could be due to different protocols, populations, methods of analysis, lack of statistic power, or methodological rigor (i.e., control group, randomization, and weight stabilization periods). Here, we performed a meta-analysis, based on data from RCTs, in order to determine the level of LBM that can be preserved when RT is associated with CR interventions in elderly obese humans. We hypothesized that elderly individuals treated with CRRT will lose less LBM compared to elderly treated with only CR.

## 2. Materials and Methods

### 2.1. Data Source

**Figure 1:**
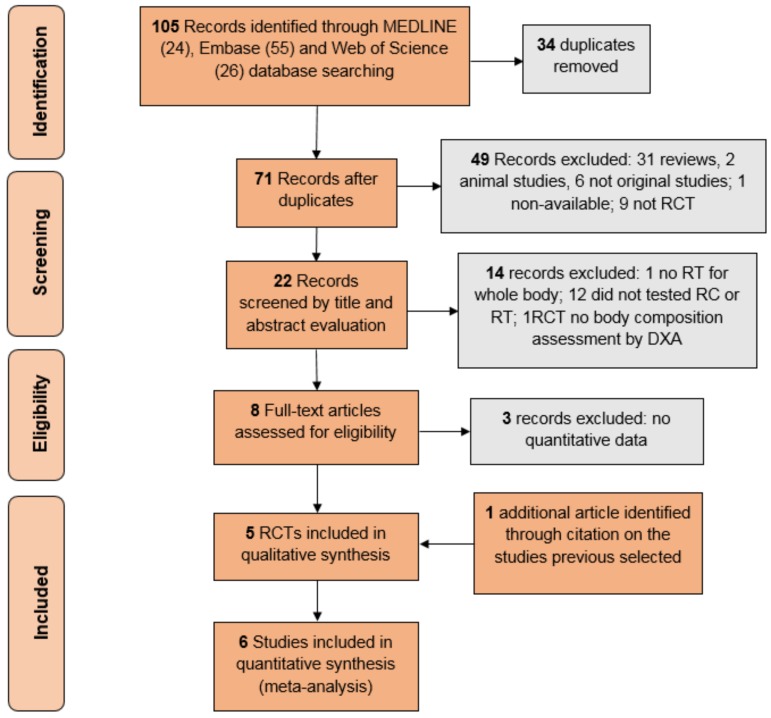
Flowchart of study selection. RCT: randomized control trials; RT: Resistance training; DXA: Dual X-ray Absorbance; CRRT: caloric restriction with resistance training group.

A systematic search was conducted between July and August 2017 using MEDLINE, Embase and Web of Science. There was no restriction on the publication’s date, and the terms were searched within all words in titles and abstracts. The following terms were searched: “caloric restriction”, “resistance training” (including “weight training”, “weight lifting”, “strength training”, “resistance exercise”, “strength exercise”, “resistance program” and “strength program”), and “muscle mass” (including “muscle body mass”, “lean mass” and “lean body mass”). Two reviewers selected the studies independently, and the disagreements were solved with further discussion. The data extraction was also made independently, and further compared to avoid errors. To isolate the effects of RT over CR, only RCTs comparing the CR with (CRRT) or without RT (CR) were included. Details of the data selection process are described in Figure 1. Non-original studies, non-human studies, and studies without a control intervention were the only exclusion criteria. Considering that only one study assessed body composition by hydrostatic weighing in young adults [21] (instead of the DEXA in older adults), and another one prescribed RT only for abdomen muscles [22] (instead of the whole body), they were not included in the meta-analyses. Six RCTs were included for the final meta-analysis [7,15,16,20,23]. One of these studies [24] reported part of its data from a previous publication [25], so both studies were included as one.

### 2.2. Study Selection

The RCTs included strictly similar samples, RT protocols, and CR protocols (Table 1). In summary, the samples were composed of older adults or elderly people (mean > 57 years old), including men, women, obese, sedentary, healthy, dyslipidemic, hyperglycemic, and diabetic individuals. Most CR diets were nutritionally balanced (55% carbohydrate, 15% protein, 30% fat) [15,16,19,20], while others had increased protein [7] or reduced fat intake [24]. Percentages of CR varied among studies, as shown in Table 1. RT protocols lasted from 12 to 24 weeks, and applied a general warm-up on a treadmill or cycle ergometer, followed by two or three trials of 8 to 15 repetitions, with a minimum of 65% of one repetition maximum (1RM) for each exercise, three times per week.

### 2.3. Assessment of Risk of Bias

The researchers assessed the studies’ qualities using the PEDro scale [26]. As patients and care providers could not be blinded in exercise interventions, these questions were nullified. Thus, scores on the PEDro scale ranged from 0 (very low methodological quality) to 9 (high methodological quality). The risk of publication bias was assessed through the Egger test.

### 2.4. Statistical Analysis

The meta-analyses were performed using Comprehensive Meta-Analysis (CMA) software, version 3.3.070. We performed three meta-analyses: lean body mass (LBM), fat body mass (FBM), and total body mass (BM). The effect size was calculated based on the raw mean difference (RMD) of the delta (pre- to post-intervention) between CRRT and control groups (CR). As the studies tested were significantly homogeneous (*p* < 0.05), the authors used the fixed effect model in all three meta-analyses. Despite the existence of particular differences between the samples studied and RT and CR protocols deserving comparisons, the absence of between-studies variance precluded further subgroup analysis. A conservative pre–post correlation of 0.5 was assumed [27]. In addition to the main results, an analysis of whole-body muscle quality was performed. The percent delta of muscle quality (strength/LBM ratio) following CRRT and CR was calculated, excluding only one study that did not report strength values [7], by the following equation: percent delta of muscle quality = (average strength/average LBM pre-intervention) − (average strength/average LBM post-intervention) × (100)/(average strength/average LBM pre-intervention). The ratio was calculated from whole-body LBM and muscle group strength presented by each original study, which count six lower limb measurements and two upper limb measurements. The Mann Whitney test was used to compare the mean differences between groups.

## 3. Results

### 3.1. Studies’ Features

The quality of studies were homogeneous, as observed by their scores of 5 [7,16,20], 6 [19,24], and 7 [15] on the PEDro scale. Egger tests (*p* > 0.1 for all) for the different analyses did not indicate any publication bias. The studies’ main features are detailed in Table 1. Some studies reported using a weight stabilization period to ensure that subjects were maintaining their weight before the intervention. All studies selected included sedentary and obese individuals, and prescribed resistance exercise for the main muscle groups, including upper and lower limbs, three times per week. LBM, FBM, and BM in kg were assessed by dual-energy X-ray absorptiometry.

### 3.2. Evidence Synthesis

**Figure 2:**
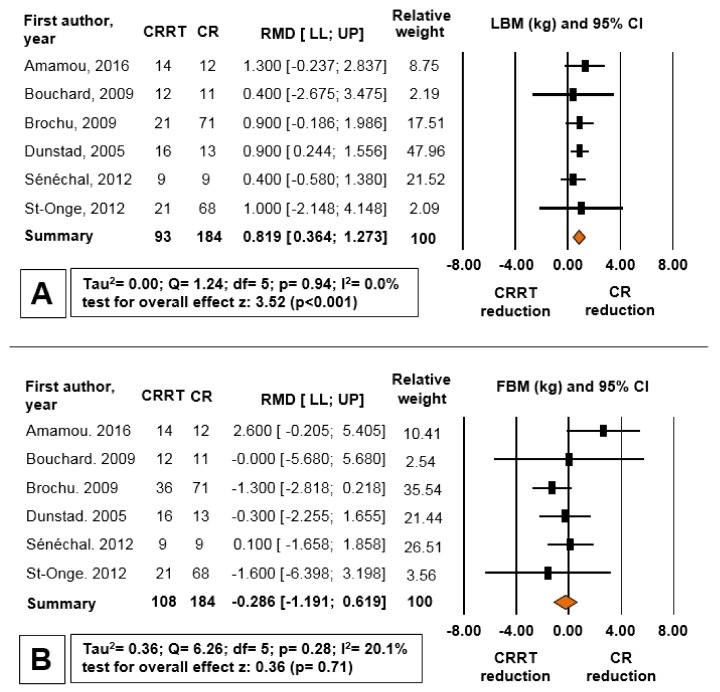

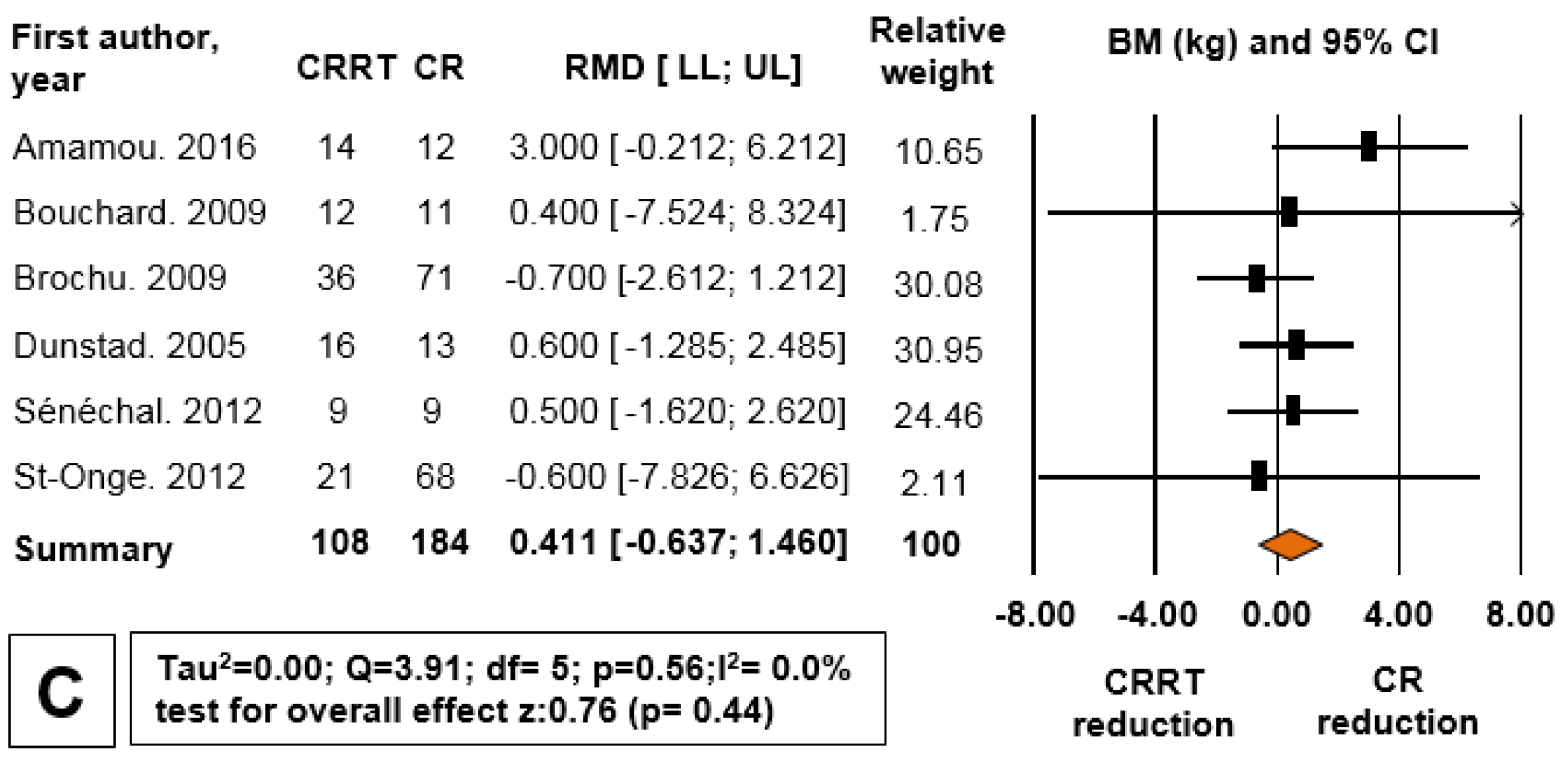
Forest plot for differences between caloric restriction plus resistance training (CRRT) and caloric restriction (CR) reductions of LBM (**A**); FBM (**B**); and BM (**C**). RMD: raw mean difference (kg); LL: lower limit of 95% CI; UP: upper limit of 95% CI; CI: confidence interval.

**Figure 3:**
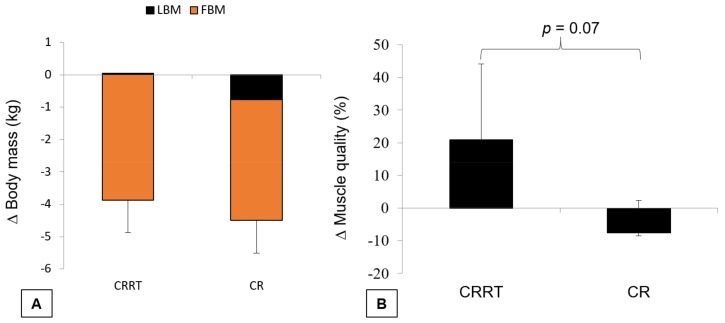
(**A**) Illustrative change in body mass after CRRT and CR; (**B**) percentage of muscle quality change after CRRT and CR. Data is presented in mean and standard deviation. P: *p*-value for difference between groups (Mann Whitney test).

Figure 2 shows the forest plot comparing the different reductions of LBM, FBM and BM for CRRT and CR. Although the reduction of BM and FBM in the CRRT group was not different from the CR group, the LBM loss in the CRRT group was 93.5% less than the CR group (RMD = 0.819 kg, 95% CI = 0.364 to 1.273, *p* < 0.001). The means standard deviations (SD) of deltas for LBM, FBM, and BM were 0.05 ± 0.3 kg, −3.86 ± 1.3 kg, and −4.16 ± 1.2 kg for CRRT, and −0.76 ± 0.1 kg, −3.73 ± 1.2 kg, and −4.73 ± 1.2 kg for CR, respectively (Figure 3a). The percentage of muscle quality changes, defined as force production per unit of muscle tissue [23], was calculated as strength divided by LBM following CRRT (20.9 ± 23.1%) and CR (−7.5 ± 9.9%), and there was a tendency for a significant difference between the groups (*p* = 0.07) (Figure 3b).

## 4. Discussion

The main finding of the present meta-analyses was that CRRT prevents 93% of the LBM loss induced by CR, although it does not affect BM and FBM reductions as compared to CR without RT. A previous meta-analysis showed only 50% LBM loss attenuation when different types of exercise were added to CR in sarcopenic obese individuals over 50 years old [10]. However, since endurance, resistance, and combined types of training were included in this analysis, it was not possible to identify which type of exercise led to the preventive effect. Endurance exercise is the most efficient type to increase energy expenditure and induce weight loss, mainly when associated with CR [28,29,30,31]. On the other hand, we propose that RT is an excellent alternative to prevent CR-induced LBM loss in elderly individuals. Furthermore, future studies should compare the preventive effect of different modalities of exercise training on CR-induced LBM loss.

A proposed mechanism to explain such protection relies on the energy costs of protein synthesis. During CR without RT, the blunted muscle protein synthesis with elevated proteolysis might allow energy maintenance [32]. Alternatively, CRRT induces muscle protein synthesis [33], likely shifting energy towards LBM maintenance while stimulating fat depletion, to allow for fuel availability to cope with the increased energy demand. Indeed, Murphy and colleagues have shown that RT restores the depressed rates of myofibrillar protein synthesis induced by CR [33].

Preservation of LBM with CR could also be obtained by additional protein intake. Longland and colleagues [34] have shown that CRRT with high protein consumption induces an increase in LBM and promotes larger fat loss, if compared to CRRT with low protein consumption. The only study that has investigated the CR effects in combination with higher protein intake (30% compared to 15% of the rest) found lower LBM loss than the others (RMD 1.3 kg compared to the main effect of RMD 0.6 kg) [7]. However, whether or ot the beneficial effects of CR are dependent on protein restriction is not clear, and deserves further attention. Although the evidence towards a longer lifespan in humans is still unclear [35], decreased protein intake is often beneficial and increases lifespan in other animals [36]. Moreover, the mammalian target of rapamycin (mTOR) pathway, which is induced by amino acids, growth factors, and RT, is often inhibited during CR, and mediates CR-induced health benefits in model organisms [37]. However, while chronic mTOR activation during obesity or aging might be deleterious [37], like in CRRT, mTOR activation concurrent with decreased energy balance may preserve protein synthesis, while stimulating fat depletion.

Cross-sectional studies have shown potential detrimental effects of higher muscle mass on insulin sensitivity in sedentary older adults [38], which oppose the well-known beneficial effects of exercise on glucose metabolism [39,40]. However, it is likely that the exercise-induced LBM increase may not lead to such impairments in insulin sensitivity [41]. While reduced glucose disposal, total cholesterol, and LDL were maintained or even improved upon implementing CRRT [7,15,16,24], in two studies blood triglyceride levels were reduced after CR, but not after CRRT [15,16]. In one of them, HDL was decreased following CRRT [15], and fasting insulin was reduced only in the CR group [16]. The authors suggest these controversial findings may be due to the presence of a varied pool of diabetic, dyslipidemic, or hypertensive patients among the study populations. These observations reinforce the need for further comparisons of CR effects and exercise training on overall health markers in healthy and diseased populations.

It goes beyond the scope of this study to determine whether CR-induced LBM reduction can be as harmful to muscle function as aging-induced LBM reduction. Sarcopenia is characterized not only by an LBM reduction, but also a reduction in muscle function [11]. Despite the fact that larger muscle areas are associated with higher muscle strength, LBM is not the only determinant [42,43]. Thus, despite the marked LBM reduction following CR in some species, CR delays age-associated muscle dysfunction in *D. melanogaster* [44] and rhesus monkeys [45], and delays the onset of sarcopenia in the latter [46].

CR intervention in elderly humans results in a reduction [18,19] or maintenance [15,16,20,24] of muscle strength. Even though the present study was not designed to test muscle function, we showed a trend towards the increase in whole body muscle quality when RT was added to CR (*p* = 0.07), suggesting that in humans, RT improves muscle function regardless of muscle mass changes during CR. It is noteworthy that muscle group strength was related to whole LBM, instead of local muscle mass, which is a limitation of the method. In agreement with our findings, in a study with elderly individuals, when CRRT was compared to RT alone, the addition of CR to RT improved mobility (400 m of walk time) without compromising other functional adaptations of RT alone [14].

Another concern regarding body composition following CR interventions is the bone mass loss, which exercise is shown to prevent, at least in rodent models of male senile osteoporosis [47]. RT is known to be highly effective to increase bone mineral density after long-term interventions in humans [48]. However, the only intervention that assessed bone mass in this review was too short to address either positive or negative effects from CRRT or CR [20]. In this sense, despite the anti-aging potential of CR to humans, future studies are required to test its long-term effects in a comprehensive health perspective.

## 5. Conclusions

CRRT almost stopped CR-induced LBM loss completely, while resulting in similar FBM and BM reductions as seen with CR alone. The confidence intervals showed there was a wide range of responsivity among individuals; therefore, future studies should investigate which factors are different between groups of responders and non-responders for LBM prevention after CRRT, in order to address the possible mediators of this process.

## Figures and Tables

**Table 1:** Studies features.

First Author, Year	Weight Stabilization	Age (Years Mean ± SD)	Sex	Health Status	CR or BM Reduction	Diet (CARBOHYDRATE/Protein/fat%)	RT Load	RT Volume	CRRT Duration (Weeks)
Amamou, 2016 [6]	4 weeks stabilization	65.8 ± 3.1	both	dyslipidemic and diabetics	472.74 ± 52.5/day	25–30 g protein supplementation (45–50/25–30/25–30)	65 to 80% 1RM	2 × (8 to 15)	16
Bouchard, 2009 [18]	no	63 ± 4	women	health	0.5 to 1 kg/week	balanced (55/15/30)	80% 1RM	3 × 8	12
Brochu, 2009 [15]	2 kg stabilization	57.2 ± 5	women	health	624 ± 133/day (33.4 ± 4.9%)	balanced (55/15/30)	65 to 75% 1RM	(2 to 3) × (15 to 10)	24
Dunstan, 2005 [21]	not reported	67.6 ± 5.2	both	diabetics	0.25 kg/week	balanced (70% carbohydrate and protein/30%fat)	75 to 85% 1RM	3 × (8 to 10)	24
Sénéchal, 2012 [14]	no	62.6 ± 4.1	women	health	0.5 to 1 kg/week	balanced (55/15/30)	not reported	3 × 8	12
St-Onge, 2012 [19]	4 weeks stabilization	57.6 ± 4	women	health	500 to 800 kcal/day	balanced (55/15/30)	8 to 15RM	(1 to 3) × (8 to 12)	24

CR: caloric restriction; BM: body mass; 1RM: one repetition maximum; RM: range of repetition maximum; HRmax: maximum heart rate predicted by age equations; BM: body mass.
